# Homelessness and health-related outcomes: an umbrella review of observational studies and randomized controlled trials

**DOI:** 10.1186/s12916-022-02423-z

**Published:** 2022-07-12

**Authors:** Michele Fornaro, Elena Dragioti, Michele De Prisco, Martina Billeci, Anna Maria Mondin, Raffaella Calati, Lee Smith, Simon Hatcher, Mark Kaluzienski, Jess G. Fiedorowicz, Marco Solmi, Andrea de Bartolomeis, André F. Carvalho

**Affiliations:** 1 Section of Psychiatry, Department of Neuroscience, Reproductive Science, and Odontostomatology, Federico Ii University of Naples, Naples, Italy; 2 Pain and Rehabilitation Centre, and Department of Medical and Health Sciences, Linköping University, SE-581 85 Linköping, Sweden; 3 Department of Psychology, University of Milan-Bicocca, Piazza dell’Ateneo Nuovo, 1, 20126 Milan, Italy; 4 Department of Adult Psychiatry, Nimes University Hospital, 4 Rue du Professeur Robert Debré, 30029 Nimes, France; 5 Cambridge Centre for Health, Performance and Wellbeing, Anglia Ruskin University, Cambridge, UK; 6 Department of Psychiatry, University of Ottawa, Ottawa, ON Canada; 7 Department of Mental Health, The Ottawa Hospital, Ottawa, ON Canada; 8 Clinical Epidemiology Program, Ottawa Hospital Research Institute, Ottawa, ON Canada; 9 Early Psychosis: Interventions and Clinical-Detection (EPIC) Lab, Department of Psychosis Studies, Institute of Psychiatry, Psychology, London, UK; 10 Faculty of Environmental and Life Sciences, Center for Innovation in Mental Health, School of Psychology, University of Southampton, Southampton, UK; 11 UNESCO staff, Chair - “Education for Health and Sustainable Development”, University of Naples, Federico II Naples, Naples, Italy; 12 IMPACT, The Institute for Mental and Physical Health and Clinical Translation, School of Medicine, Barwon Health, Deakin University, Geelong, Australia

**Keywords:** Homeless, Health outcomes, Severe mental illness, Umbrella review

## Abstract

**Background:**

Homelessness has been associated with multiple detrimental health outcomes across observational studies. However, relatively few randomized controlled trials (RCTs) have been conducted on people who experience homelessness (PEH). Thus, this umbrella review ranked the credibility of evidence derived from systematic reviews (SRs) and meta-analyses (MAs) of observational studies investigating the associations between homelessness and any health outcome as well as RCTs targeting health needs in this population.

**Methods:**

Several databases were systematically searched from inception through April 28, 2021. Any SR and/or MA reporting quantitative data and providing a control group were eligible for inclusion. The credibility of the evidence derived from observational studies was appraised by considering the significance level of the association and the largest study, the degree of heterogeneity, the presence of small-study effects as well as excess significance bias. The credibility of evidence was then ranked in five classes. For SRs and/or MAs of RCTs, we considered the level of significance and whether the prediction interval crossed the null. The AMSTAR-2 and AMSTAR-plus instruments were adopted to further assess the methodological quality of SRs and/or MAs. The Newcastle-Ottawa Scale (NOS) was employed to further appraise the methodological quality of prospective cohort studies only; a sensitivity analysis limited to higher quality studies was conducted.

**Results:**

Out of 1549 references, 8 MAs and 2 SRs were included. Among those considering observational studies, 23 unique associations were appraised. Twelve of them were statistically significant at the *p*≤0.005 level. Included cases had worst health-related outcomes than controls, but only two associations reached a priori-defined criteria for convincing (class I) evidence namely hospitalization due to any cause among PEH diagnosed with HIV infection, and the occurrence of falls within the past year among PEH. According to the AMSTAR-2 instrument, the methodological quality of all included SRs and/or MAs was “critically low.” Interventional studies were scant.

**Conclusion:**

While homelessness has been repeatedly associated with detrimental health outcomes, only two associations met the criteria for convincing evidence. Furthermore, few RCTs were appraised by SRs and/or MAs. Our umbrella review also highlights the need to standardize definitions of homelessness to be incorporated by forthcoming studies to improve the external validity of the findings in this vulnerable population.

**Supplementary Information:**

The online version contains supplementary material available at 10.1186/s12916-022-02423-z.

## Background

Homelessness is an important social, public health, and human rights issue worldwide. The prevalence of homelessness varies among diverse countries and cultures around the world. Lifetime prevalence estimates from representative samples are 4.2% in the USA [1] to 4.9% in Europe [2]. However, high-quality data on the prevalence of homelessness in low- and middle-income countries (LMICs) is scant.

The operational definitions for homelessness likewise vary across different literature sources and settings [3] although a commonly accepted and implemented definition of homelessness globally comes from the European Typology of Homeless and Housing Exclusion study [4].

People experiencing homelessness (PEH) may face social and economic challenges that may lead to poor health, such as poverty, poor nutrition, and social exclusion. People who lack stable and appropriate housing appear to be at relatively high risk for a broad range of acute and chronic illnesses, especially infectious diseases, heart diseases, substance use disorders, and severe mental disorders [5]. However, it is unclear whether homelessness causes these disorders or otherwise these illnesses per se contribute to homelessness. Finally, evidence indicates that PEH has a lower probability of receiving proper care for their health conditions compared to the general population [6].

Data about differences in the prevalence of multiple health conditions between PEH and the general population is substantially unreliable, as exemplified by current knowledge about mental health [7] and infectious diseases among PEH [8]. Cohort and case-control studies have reported various health outcomes associated with homelessness, and several health outcomes have been the subject of a multitude of systematic reviews (SRs) and meta-analyses (MAs). While informative, this latter knowledge synthesis is usually restricted to a single outcome, and some of their results may be affected by biases, which are often poorly appraised [9]. Furthermore, randomized controlled trials (RCTs) targeting health-related outcomes in homeless populations are few, thus providing limited evidence to inform health policies [10]. Specifically, significant associations claimed by the original observational studies, or their pooled synthesis may be susceptible to biases such as excess significance [11], publication bias, reporting bias, and residual confounding, leading to misleading or inflated estimates of these associations [12].

Umbrella reviews (URs)—a systematic collection and appraisal of SRs and MAs performed on a specific topic [13]—can disentangle the aforementioned biases through appraising the quality and comprehensiveness of the data, and hence, assess which associations derived from observational studies are supported by the most credible evidence. Likewise, URs can provide a methodological appraisal of RCTs targeting a specific population or condition. Thus, in the current report, we aimed to conduct an umbrella review of the evidence from observational studies and RCTs considering multiple health outcomes involving PEH. In particular, we aimed at (i) assessing the reported association measures between homelessness and any health outcome and (ii) appraising the interventions targeting any health outcome among PEH.

## Methods

### Search strategy

We performed an umbrella review that included observational or RCTs that investigated the association between homelessness and any health outcome. The PubMed/MEDLINE, EMBASE, and SCOPUS databases were systematically searched from inception up to April 28, 2021. The following string was adopted for PubMed: (((“homeless persons”[MeSH Terms]) OR (“homeless youth”[MeSH Terms])) OR (“vulnerable populations”[MeSH Terms]) OR (homeless*[Title/Abstract])) AND (((((“meta analysis as topic”[MeSH Terms]) OR (“systematic reviews as topic”[MeSH Terms])) OR (“meta analysis”[Title/Abstract])) OR (“systematic review”[Title/Abstract]))). Please see Additional file 1: material 1. The definition of homelessness and related phenomena were independently recorded by two investigators.

### Eligibility criteria

For the synthesis of evidence from SRs and MAs of observational studies, we included those studies reporting any health outcome among PEH compared to the general population or otherwise provided controls (i.e., people who are not experiencing homelessness, PEH without a particular exposure). We excluded those SRs or MAs of observational studies that only provided prevalence estimates of a given health condition in PEH without providing a measure of association. Specifically, those studies reporting interventions just targeting housing, but not the related health status, were likewise excluded. The SRs and MAs of RCTs suitable for inclusion were those documenting interventions targeting any health outcome among PEH; controls were PEH exposed to a health-targeting intervention different from the health-outcome intervention delivered to PEH cases. Eligible quantitative SRs and MAs of observational studies had to include at least 3 studies; eligible SRs and MAs of intervention studies had to include at least 5 studies. The rationale for this inclusion criterion is explained in detail elsewhere [14]. In the case of multiple MAs reporting on the same topic (i.e., overlapping with the same type of intervention or exposure), we considered only the one that included the largest number of studies as it is a standard procedure in previously conducted umbrella reviews [15, 16]. In cases there were two or more MAs pooling the same number of studies, we retained the most recent one. Qualitative reports were excluded. There were no language restrictions for the inclusion of studies for this umbrella review.

The protocol for this study was registered in PROSPERO with the following numbers: CRD42021252185, for the protocol investigating observational studies, and CRD42021252191 for the protocol that assessed evidence from intervention studies, respectively. Complete versions of each protocol are fully available online at https://osf.io/am67d/ and https://osf.io/58mhu/.

### Data extraction

Three investigators (MDP, MB, MF) independently searched title/abstracts of retrieved references for eligibility, and when a consensus could not be achieved, additional authors with considerable expertise in umbrella reviews (AFC, MS, LS) and the study of homelessness (LS, JGF, SH, MK) were consulted. The same procedure was followed at the full-text level. The reference lists of included studies were also searched for the identification of additional eligible references. Among other variables, we recorded the following: publication year, considered health outcome, study design, number of the included studies, total sample size, homelessness definition, and disclosure of sponsorship. For each primary study included in the SRs or MAs, we additionally recorded the first author, year of publication, study design (i.e., cohort, case-control, cross-sectional, RCT), setting of the study (i.e., inpatients, outpatients, population-based), number of subjects included in the study (total sample, cases, and controls), sex, ethnicity, both adjusted and unadjusted effect sizes (ES), and 95% confidence interval (CI). Intervention and mean duration of RCT studies were likewise extracted.

### Data analysis and assessment of the credibility of evidence

We re-analyzed each eligible MA using the extracted individual study estimates to compute the summary effect estimates and the exact p-value under the random-effects model with DerSimonian and Laird method if included studies were equal or more than 10, and Hartung, Knapp, Sidik, and Jonkman (HKSJ) if less than 10 [17, 18]. Cochran’s *Q* test and the *I*^2^ statistics were computed for the evaluation of heterogeneity across studies (*I*^2^>50% indicated high heterogeneity) [19, 20]. To further account for heterogeneity between studies, we computed 95% prediction intervals for the summary random-effect estimates [21]. We evaluated the presence of small-study effects (i.e., large studies fetching significantly more conservative results than smaller studies) by adopting the Egger’s regression asymmetry test (*p*≤0.10) [22]. For statistically significant MAs, we assessed the presence of excess significance bias by evaluating whether the number of observed studies with nominally statistically significant results was different from the expected number of studies with statistically significant results [23]. The expected number of statistically significant studies in each association was calculated from the sum of the statistical power estimates for each component study using an algorithm from a non-central t distribution [24–26]. The power estimates of each component study depended on the plausible effect size of the tested association, which was assumed to be the effect size of the largest study in each MA [27]. The presence of excess significance bias for individual MAs was considered at *p*≤0.10. The credibility of the evidence of each association provided by MAs of observational studies was assessed using the criteria previously applied in various medical fields [26, 28, 29], waiving the “number of cases” criterion since some health outcomes of PEH represent infrequent events. Briefly, the associations that presented nominally significant random-effect summary estimates were considered as “convincing” (Class I), “highly suggestive” (Class II), “suggestive” (Class III), “weak evidence” (Class IV), or “non-significant” (NS). Please, see the credibility box in Additional file 1: Table S1. For MAs of intervention studies, we assessed the significance of the pooled effect size as *P*<0.005, *P*=0.005–0.005, and *P*≥0.05 [30], the 95% prediction interval (excluding the null or not), the significance of the effect size of the largest study, and the presence of large heterogeneity (i.e., *I*^2^>50%) [31]. In addition, the methodological quality of those SRs and/or MAs was further appraised with the Assessment of Multiple Systematic Reviews Plus (AMSTAR-Plus) instrument [32]. All statistical tests were two-tailed. The data abstraction was performed using pre-defined Microsoft Excel® forms, while the statistical computations were carried out by an expert senior author (ED) using the STATA/SE, version 17.0 (StataCorp LLC) software. For each eligible quantitative report, two investigators (MDP and MB) independently rated the methodological quality using the AMSTAR-2 (Assessment of multiple SRs) tool [33] for quantitative SRs or MAs of observational studies. The prospective cohort studies included in the quantitative SRs or MAs of observational studies were rated for quality using the Newcastle-Ottawa scale (NOS) [34]. Finally, we adopted the following thresholds for the NOS scores: “good quality” (3-4 stars in the “selection domain” AND 1-2 stars in the “comparability” domain AND 2-3 stars in the “outcome” domain), “fair quality” (2 stars in the “selection domain” AND 1-2 stars in the “comparability” domain AND 2-3 stars in the “outcome” domain), and “poor quality” (0-1 stars in the “selection domain” OR 0 stars in the “comparability” domain OR 0-1 star in the “outcome” domain) [35].

## Results

**Fig. 1:**
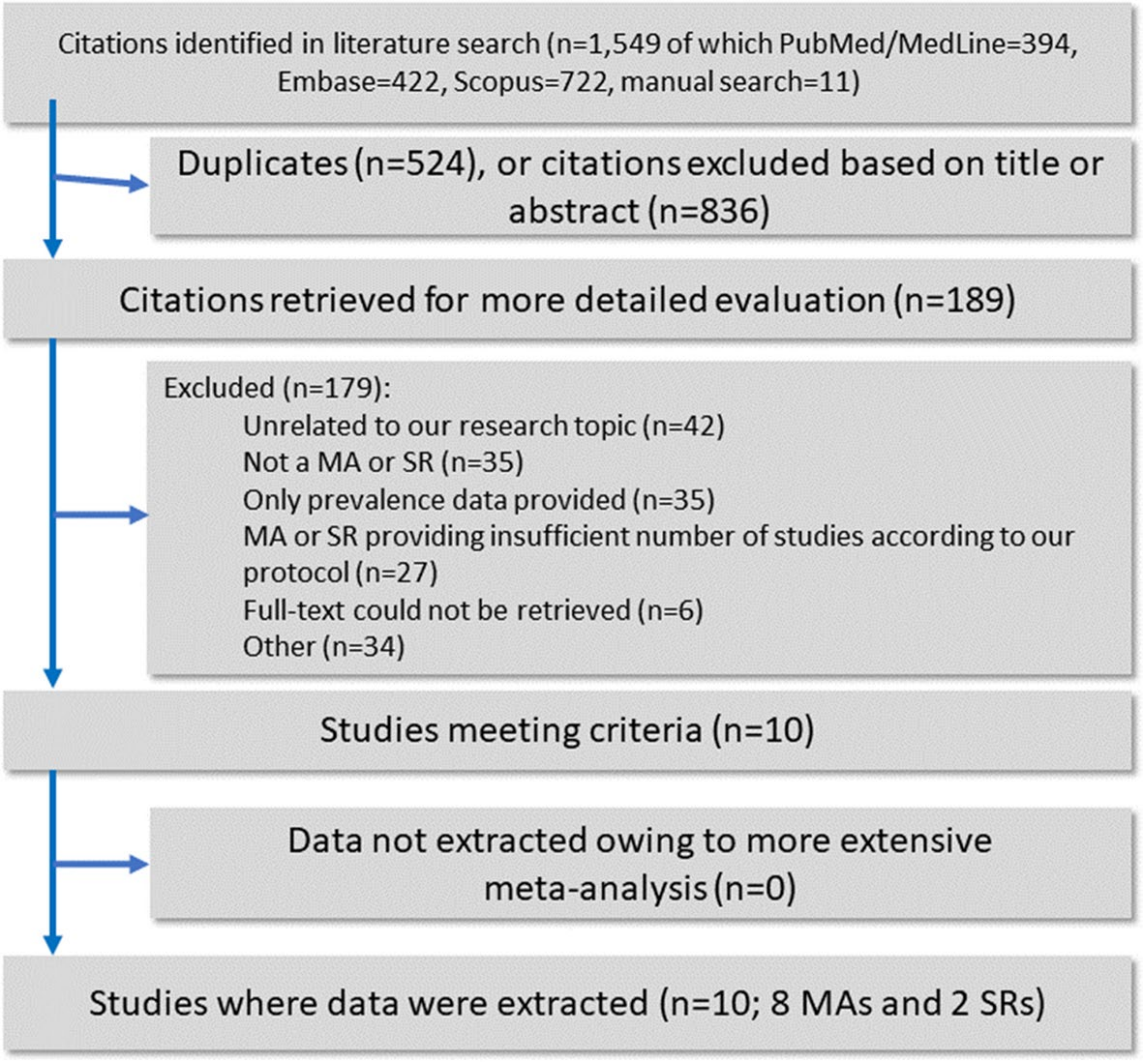
Flowchart of the literature search and evaluation process returning 10 systematic reviews and meta-analyses

The search returned 1549 potentially eligible records, of which 11 records were manually retrieved. Upon title and abstract screening, 189 records were further assessed at the full-text level, of which 179 were excluded with reasons as detailed in Additional file 1: Table S2 [7, 8, 36–210]. Nine SRs or MAs of observational studies [211–219] were included, which yielded 23 comparisons. One MA of interventional studies [220] fetched two comparisons. Figure 1 provides a flowchart for study selection.

Given the scant evidence for intervention studies, we decided to combine the reporting of both registration protocols into a single publication.

Descriptive characteristics of the 10 included eligible SRs and MAs of observational and intervention studies are outlined in Table 1. The observational studies had the following control groups: PEH without SUD [211], PEH who did not inject drugs [212], people not experiencing homelessness [213–215, 218, 219], or the general population [216, 217]. Although substantially overlapping, we nonetheless recorded “people not experiencing homelessness” and “general population” controls, as documented by the original study at review. The included MA of RCTs controlled for various treatment exposures [220].

**Table 1:** Characteristics of the included SRs or MAs of observational and interventional studies

Author, year (type of study)	Type of studies included	Health outcome examined	Exposures/active treatment	Control group	No. of studies included in the MA or SR (No. of studies included in the present UR)	Population	Homelessness adopted definition	AMSTAR-2 or AMSTAR-plus
Tweed et al., 2021 (MA) [215]	Cross-sectional, case-control, cohort studies, and baseline data from interventional studies	Mortality, morbidity, health-related quality of life, self-rated health	Homelessness, imprisonment, substance use (other than alcohol, cannabis, or performance-enhancing drugs), sex working, SMI	Absence of homelessness, imprisonment, substance use, sex working, or SMI	237 (5)	People experiencing homelessness, imprisonment, substance use, sex working, or SMI	People who are rough sleeping or unstably/marginally housed	Critically low
Arum et al., 2021 (MA) [216]	Cross-sectional, case-control, cohort studies	HIV and HCV diagnosis	Homelessness, unstable housing	Absence of homelessness or unstable housing	37 (24)	People who use injective drugs	Lacking access to adequate housing, according to the Institute of Global Homelessness	Critically low
Suh et al., 2020 (MA) [217]	Cross-sectional, cohort studies	Geriatric syndrome outcomes	Homelessness	Absence of homelessness	5 (4)	People experiencing homelessness	People lacking a fixed, regular, and adequate nighttime residence, including those utilizing temporary shelters, being homeless, living in an abandoned building or vehicle, or any other unstable or nonpermanent situation, according to the US Department of Health and Human Services	Critically low
Al-Shakarchi et al., 2020 (MA) [218]	Case-control, cohort studies	Cardiovascular disease	Homelessness	Absence of homelessness	17 (9)	People experiencing homelessness	Not provided	Critically low
van Draanen et al., 2020 (SR) [219]	Cross-sectional, case-control, cohort studies	Opioid-related fatal and non-fatal overdose	Any measure of socioeconomic marginalization	Different levels of socio-economic marginalization	37 (4)	People who use opioids in North America, Europe, the United Kingdom, Australia, and New Zealand	Not provided	Critically low
Lin et al., 2019 (MA) [220]	Cross-sectional, cohort studies	Antiretroviral therapy adherence	Homelessness, sex working, or drug use	Absence of homelessness, sex working, or drug use	29 (4)	People with HIV infection	Not provided	Critically low
Aldridge et al., 2017 (MA)	SRs, MAs, cross-sectional, cohort studies	Mortality, morbidity	Homelessness, imprisonment, sex working, substance use disorder	Absence of homelessness, imprisonment, sex working, or substance use disorder	337 (3)	People experiencing homelessness, imprisonment, substance use, or sex working	Not provided	Critically low
Bassuk et al., 2015 (MA) [222]	Cross-sectional, case-control, cohort studies	Mental health disorders, behavioral disorders	Homelessness	Absence of homelessness	7 (7)	Children/adolescents experiencing homelessness aged less than 18 years, enrolled in the United States and accompanied by a parent	The literal definition of homelessness includes emergency shelter, transitional housing, residing in places not meant for human habitation, fleeing domestic violence, or not having an identified residence	Critically low
Aidala et al. 2016 (SR) [223]	Cross-sectional, case-control, cohort studies, RCT	HIV health care access and utilization, adherence to antiretroviral treatments, HIV clinical health outcomes, other health outcomes, emergency department, and inpatient use, HIV risk behaviors	Homelessness, unstable housing	Absence of homelessness or unstable housing	152 (19)	People with HIV infection who live in high-income countries	Not provided	Critically low
Hyun et al., 2020 (MA) [224]	RCT, cluster RCT	Depression, anxiety, mental health status, PTSD symptoms, psychological distress, self-efficacy, quality of life	Psychosocial interventions	Control condition	11 (6)	People experiencing homelessness	Living situation of rooflessness without a shelter of any kind and houselessness with a temporary institution or shelter to sleep, according to the European typology on homelessness and housing exclusion	10

*MA* meta-analysis, *PTSD* post-traumatic stress disorder, *RCT* randomized controlled trial, *SMI* severe mental illness, *SR* systematic review, *UR* umbrella review

### Description and summary of associations

#### Observational studies

**Fig. 2:**
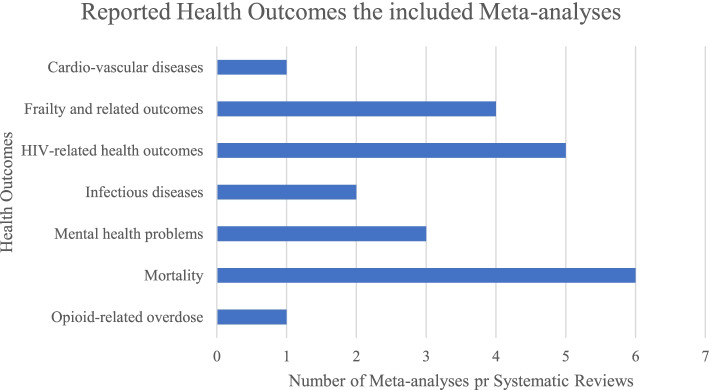
Number of systematic reviews or meta-analyses of observational studies reporting health outcomes among PEH by category of health outcome

Nine eligible SRs and/or MAs of observational studies assessed 23 associations, evaluated by 122 individual studies from 73 original reports, estimating adverse health outcomes associated with homelessness. Six (26.1%) associations concerned various causes of mortality among PEH, five (21.7%) associations regarded health outcomes related to HIV infection, and four (17.4%) associations inquired about premature geriatric syndromes (e.g., falls, functional limitations). Please refer to Fig. 2 for additional details. Twelve out of 23 (52.2%) associations were nominally statistically significant at a *p*≤0.005 level based on the random-effects model, and 7 (30.4%) reached *p*≤1×10^−6^. Fifteen associations (65.2%) had large heterogeneity, and the 95% prediction interval excluded the null value for only five associations (21.7%). In twenty associations (86.9%), the effect of the largest study was statistically significant at *p*≤0.05. A small-study effect was detected in one association (4.3%), and excess significance bias occurred in one out of ten studies suitable for such estimation (10%). Please refer to Table 2 for details.

**Table 2:** Evidence from SRs or MAs of observational studies for the association between homelessness and any health outcome

							Criteria for Level-of-Evidence Classification						
Author, year	Adverse health outcomes	Exposed/unexposed	Prevalence (%) based on cohort studies	No. of included studies per association	Random-effects measure, ES (95% CIs)	Results	***p*** value random effects	***I***^**2**^ (***p*** value)	PIs (95 % CIs)	SSE/ESB	LS	CE	AMSTAR-2 quality
Aidala et al., 2016 [223]	Hospitalization due to any cause	PEH with HIV/Non-homeless with HIV	21.8%	4	OR: 2.05 (1.7–2.46)	Increased odds for PEH with HIV	4.6×10^−15^	4.5% (0.85)	1.31–3.19	No/no	Yes	I	Critically low
Suh et al., 2020 [217]	Falls in the previous year	PEH/Non-homeless	NA	3	RR: 3.42 (3.15–3.70)	Increased chances for PEH	1.07×10^−199^	0.08% (0.969)	2.03–5.73	No/No	Yes	I	Critically low
Aldridge et al., 2017	Mortality due to any cause	PEH/General population	NA	5	SMR: 6.22 (4.2–9.2)	Increased chances for PEH	6.8×10^−20^	98.16% (0.0001)	1.53–25.33	No/NA	Yes	II	Critically low
Aldridge et al., 2017	Mortality due to external causes, as defined by ICD-10	PEH/General population	NA	6	SMR: 15.75 (10.58–23.44)	Increased chances for PEH	5.2×10^−42^	97.43% (0.0001)	3.63–68.17)	No/NA	Yes	II	Critically low
Arum et al., 2021 [216]	HCV infection	PEH who inject drugs/PEH who did not inject drugs	NA	19	RR: 1.66 (1.37−2)	Increased chances for PEH who inject drugs	1.4×10^−7^	55.4% (0.0019)	0.88–3.12	No/NA	Yes	II	Critically low
Suh et al., 2020 [217]	Limitations in activities of daily living	PEH/Non-homeless	NA	4	RR: 1.46 (1.27–1.68)	Increased chances for PEH	8.7×10^−8^	53.2% (0.057)	0.85–2.49	No/No	Yes	II	Critically low
Suh et al., 2020 [217]	Limitations in instrumental activities of daily living	PEH/Non-homeless	NA	4	RR: 1.36 (1.25–1.48)	Increased chances for PEH	7.4×10^−13^	44.9% (0.199)	0.99–1.85	No/No	Yes	II	Critically low
Aidala et al., 2016 [223]	Emergency department use due to any cause	PEH with HIV/Non-homeless with HIV	21.6%	6	OR: 1.73 (1.31–2.28)	Increased chances for PEH with HIV	1×10^−4^	67.2% (0.22)	0.75–3.97	No/No	Yes	III	Critically low
Aidala et al., 2016 [223]	Nonadherence to antiretroviral therapy	PEH/Non-homeless	8.5%	5	OR: 1.55 (1.29–1.86)	Increased chances for PEH	2.3×10^−6^	16.5% (0.689)	1.04–2.31	No/Yes	Yes	III	Critically low
Al-Shakarchi et al., 2020 [218]	Diagnosis of cardiovascular disease, as defined by ICD-10	PEH/Non-homeless	26.8%	9	OR: 2.59 (1.61–4.18)	Increased chances for PEH	9.6×10^−5^	98.4% (0.0001)	0.44–15.23	No/NA	Yes	III	Critically low
Arum et al., 2021 [216]	HIV infection	PEH who inject drugs/PEH who did not inject drugs	NA	12	RR: 1.43 (1.12–1.83)	Increased chances for PEH who inject drugs	0.0037	52.9% (0.015)	0.72–2.85	No/NA	Yes	III	Critically low
van Draanen et al., 2020 [219]	Opioid-related overdose	PEH/Non-homeless	NA	4	OR: 2.10 (1.43–3.10)	Increased chances for PEH	1.5×10^−4^	59.8% (0.032)	0.45–9.75	No/NP	Yes	III	Critically low
Bassuk et al., 2015 [222]	Mental Health problems, assessed by the Child Behavior Checklist	Homeless schoolchildren/non-homeless school children	NA	3	OR: 1.77 (1.13–2.76)	Increased chances for Homeless school children	0.012	17.9% (0.518)	0.05–64.4	No/NP	Yes	IV	Critically low
Lin et al., 2019 [220]	Adherence to antiretroviral therapy	PEH/General population	NA	4	OR: 0.5 (0.32–0.77)	Better adherence in the general population	0.002	72.4% (0.064)	0.08–3.16	No/No	Yes	IV	Critically low
Suh et al., 2020 [217]	Frailty	PEH/Non-homeless	NA	3	RR: 2.59 (1.05–6.39)	Increased chances for PEH	0.0388	97.8% (0.0001)	0.001–2.8×10^5^	Yes/NA	Yes	IV	Critically low
Tweed et al., 2021 [215]	Mortality due to external causes, as defined in ICD-10	PEH with SUD/PEH without SUD	NA	4	HR: 2.3 (1.26–4.2)	Increased chances for PEH with SUD	0.0069	78.5% (0.093)	0.17–31.02	No/NA	Yes	IV	Critically low
Aidala et al., 2016 [223]	Mortality due to any cause	PEH with HIV/Non-homeless with HIV	32.8%	7	HR: 1.43 (0.74–2.77)	Increased chances for PEH with HIV	0.286	90.5% (0.001)	0.15–13.69	No/No	Yes	NS	Critically low
Aidala et al., 2016 [223]	Viral load non-suppression	PEH with HIV/Non-homeless with HIV	5.6%	3	OR: 1.23 (0.89–1.71)	Increased chances for PEH with HIV	0.215	70% (0.083)	0.03–51.48	No/No	No	NS	Critically low
Bassuk et al., 2015 [222]	Mental Health problems, assessed by the Child Behavior Checklist	Homeless pre-school children/non-homeless pre-school children	4.6%	3	OR: 1.47 (0.93–2.35)	Increased chances for Homeless pre-school children	0.101	13.9% (0.544)	0.04–53.39	No/NP	Yes	NS	Critically low
Bassuk et al., 2015 [222]	Mental Health problems, assessed by the Child Depression Inventory	Homeless school children/non-homeless school children	NA	3	OR: 1.45 (0.77–2.73)	Increased chances for Homeless school children	0.248	31.6% (0.35)	0.01–453.76	No/NP	No	NS	Critically low
Tweed et al., 2021 [215]	Mortality due to any cause	PEH with SUD/PEH without SUD	45.5%	5	HR: 1.6 (0.99–2.57)	Increased chances for PEH with SUD	0.0536	96.1% (1×10^−4^)	0.26–9.61	No/No	Yes	NS	Critically low
Tweed et al., 2021 [215]	Mortality due to any cause	PEH with SMI/PEH without SMI	8.4%	3	HR: 0.89 (0.69–1.15)	Increased chances for PEH without SMI	0.382	19.9% (0.473)	0.1–7.64	No/NP	No	NS	Critically low
Tweed et al., 2021 [215]	Mortality due to external causes, as defined by ICD-10	PEH with SMI/Homeless without SMI	NA	3	HR: 3.13 (0.78–12.51)	Increased chances for PEH with SMI	0.106	93.4% (0.003)	0.001–9.6×10^7^	No/NA	Yes	NS	Critically low

*CE* credibility evidence, *CI* confidence interval, *ES* effect size, *ESB* excess significance bias, *HR* hazard ratio, *ICD-10* International Classification of Diseases, 10th revision, *LS* largest study with significant effect, *MA* meta-analysis, *NA* not applicable, *NP* not pertinent because of fewer than expected number of observed studies, *NS* not significant, *OR* odds ratio, *PEH* people experiencing homelessness, *PI* prediction interval, *RR* risk ratio, *SMI* severe mental illness, *SMR* standardized mortality ratio, *SR* systematic review, *SSE* small study effect, *SUD* substance use disorder

#### Intervention studies

One eligible MA of intervention studies documented two therapeutic interventions, evaluated by 10 unique RCTs from 10 original reports, estimating interventions associated with mental health status among PEH [220]. One association concerned the psychological interventions among PEH diagnosed with depression, while the remaining one dealt with psychological interventions for anxiety. None of the assessed associations reached a statistically significant value at *p*≤0.005 based on the random-effects model [30]. The degree of heterogeneity of the documented associations was quantified in *I*^2^=42.5% for depression and 39.9% for anxiety. The 95% prediction intervals crossed the null for the outcomes of both interventions. Neither of the largest studies of the two associations was statistically significant at *p*≤0.05. Please refer to Table 3 for details.

**Table 3:** Evidence from SRs or MAs of intervention studies for the association between different interventions for PEH and any health outcome

Author, year	Considered health outcome	Active treatment/control treatment	Number of patients allocated to each treatment	No. of included studies per association	Random-effects measure, ES (95% CIs)	Results	***p*** value random effects	***I***^**2**^ (***p*** value)	PIs (95 % CIs)	LS	AMSTAR-plus
Hyun et al., 2020 [224]	Depression	Psychological interventions/treatment as usual	236/224	5	SMD: − 0.24 (− 0.49–0.02)	Psychological interventions are more effectiver than treatment as usual	0.07	42.6% (0.2)	− 0.964–0.491	No	10
Hyun et al., 2020 [224]	Anxiety	Psychological interventions/treatment as usual	229/215	5	SMD: − 0.25 (− 0.51–0)	Psychological interventions are moe effective than treatment as usual	0.05	39.9% (0.28)	− 0.967–0.459	No	10

*CI* confidence interval, *ES* effect size, *LS* largest study with significant effect, *MA* meta-analysis, *PI* prediction interval, *RR* risk ratio, *SMD* standardized mean difference, *SR* systematic review

### Grading of systematic reviews and meta-analyses of observational studies

Concerning the SRs and/or MAs of observational studies, none of them concurrently reached a “convincing evidence” threshold, according to the adapted credibility box, and a “high quality” score based on the AMSTAR-2 tool. According to the latter, every SR and/or MA included in the present study was rated as having “critically low” methodological quality. Please refer to Additional file 1: Table S3 [211–219].

#### Convincing evidence

Among the 23 associations, two (8.7%) were supported by “convincing evidence”: hospitalization due to any cause among PEH diagnosed with HIV infection and the occurrence of falls within the past year among PEH. Both health outcomes were more common among PEH compared to non-homeless controls.

#### Highly suggestive evidence

Five (21.7%) associations were rated “highly suggestive evidence”: (1) mortality due to any cause; (2) mortality due to external causes (i.e., intentional injury, unintentional injury, poisoning) among PEH compared to the general population; (3) HCV-infection among PEH using injection drugs compared to those who did not; (4) the presence of limitations in activities of daily living (ADL, e.g., dressing, eating, toileting); and (5) instrumental activities of daily living (IADL, e.g., using telephone, using transportations, taking medications) among PEH compared to non-homeless controls. These above-mentioned health outcomes were more common among PEH compared to their respective controls. Mortality due to any cause and mortality due to external causes had high standardized mortality ratios=6.22 (95% C.I.=4.2–9.2), and SMR=15.75 (95% C.I.=10.58–23.44), respectively.

#### Suggestive, weak, and no evidence

Five (21.7%) associations were rated “suggestive evidence,” four (17.4%) were “weak evidence,” while “no significant evidence” was found in seven (30.5%) associations.

### Grading of systematic reviews and meta-analyses of intervention studies

Concerning intervention studies, the sole MA retrieved obtained a score of “10” at the AMSTAR-plus. Please refer to Table 3 for details.

### Sensitivity analysis

A sensitivity analysis limited to high-quality prospective cohort studies included eleven (47.8%) associations, rated according to the NOS. Upon sensitivity analysis, two associations worsen, and one association improved in terms of credibility evidence. Hospitalization due to any cause among PEH with HIV infection shifted from “convincing evidence” to “highly suggestive evidence”, non-adherence to antiretroviral therapy (ART) among PEH with HIV infection shifted from “suggestive evidence” to “weak evidence”, and mortality due to any cause among PEH with SUD up-graded from “no significant evidence” to “highly suggestive evidence”. Please refer to Additional file 1: Table S4 [211, 214, 217, 219].

## Discussion

### Statement of principal findings

We found convincing evidence that all-cause hospitalization in people with HIV infection and the occurrence of falls within the past year were more common among PEH compared to comparison populations. We also found highly suggestive evidence that mortality due to any cause; mortality due to external causes; hepatitis C infection among PEH using injection drugs; limitations in activities of daily living; and limitations in instrumental activities of daily living were significantly more common in PEH compared to their comparison populations. Mortality due to any cause and mortality due to external causes had high standardized mortality ratios such that PEH had a mortality rate six times their comparison groups and they were about 15 times more likely to die from either accidents or intentional self-harm.

### Strengths and limitations

To our knowledge, this study is the first umbrella review that systematically inquired about any health-related outcomes and interventions among PEH, grading the evidence by using previously adopted and widely accepted criteria of credibility [14, 16, 29, 31, 221]. All SRs/or MAs of observational studies were graded as having “critically low” methodological quality according to the AMSTAR-2 instrument. Among the associations rated with “highly suggestive evidence”, two were not deemed as “convincing” due to high heterogeneity. Overall, 65.2% of the associations covered by the present umbrella review were hampered by high heterogeneity, which held upon controlling for high-quality prospective studies (seven out of eleven associations—63.6%—had an *I*^2^>50%).

The differences in definitions of “homelessness” and thus categories of homelessness (for example individuals without permanent housing who may live on the streets; stay in a shelter, mission, single room occupancy facilities, abandoned building or vehicle; or in any other unstable or non-permanent situation) adopted by the authors of the included SRs or MAs could likewise account for the high rates of heterogeneity. Half of the appraised studies lacked a “homelessness” definition, and the remaining half provided a broad definition, as detailed in Table 1. The timeframe for the homelessness definition, or its related labels, varied across the individual studies included by the appraised SRs or MAs, often merging people who were currently homeless with people who were experiencing this condition within varying timeframes (i.e., 30-day, 6-month, or 12-month intervals, as usually documented by the authors of the original studies). Future research should, therefore, rely on consistent operational definitions, or otherwise stratify their results accordingly, especially considering that substantial variability of the adopted definitions exists across different world regions [222].

The limitations of this review include the exclusion of RCTs on the impact of housing interventions (Housing First) on PEH. However, such interventions were not deemed eligible for inclusion according to our a priori criteria since we focused on those interventions directly targeting health outcomes in PEH rather than on interventions aimed at reducing the burden of homelessness. Sensitivity analyses were restricted to high-quality prospective studies. Because of the lack of relevant SRs or MAs, we could not appraise otherwise relevant associations between homelessness and health outcomes such as alcohol-related issues, cancer, or infectious diseases other than HIV or HCV. Mental illness-related issues were only accounted for by three comparisons focusing on children experiencing homelessness [223].

### Comparison with previous studies

Considering the two associations reaching “convincing evidence” before sensitivity analysis, the documented hospitalization trend due to any cause in people with HIV is consistent with a recent retrospective cohort study [224]. A significant increase in “falls during the previous year” may be the result of high rates of concurrent geriatric syndromes, alcohol use disorders, and drug abuse as described elsewhere [225].

Hence, upstream (e.g., poverty, poor nutrition, barriers to healthcare, and HIV treatment) and downstream factors (i.e., comorbidities and multimorbidity) significantly affect PEH [5], jeopardize their healthcare, and inflate their hospitalization rates compared to the general population [222].

Although we were unable to include any SR and MA reporting on the relationship between the COVID-19 pandemic and homelessness, PEH could be at higher risk also at developing hospitalization or fatalities due to COVID-19 according to recent evidence [226] although this finding deserves replication, providing evidence for the need of well-designed interventions targeting this vulnerable population.

## Conclusions and implications for further research

This review adds weight to arguments about why reducing homelessness should be a priority beyond human rights justification. The evidence that experiencing homelessness leads to worse health outcomes is only a secondary consideration for providing affordable housing albeit an important one. Housing reduces hospitalization rates according to RCTs involving PEH with chronic illnesses [227, 228]. This study demonstrates that a readily treatable illness such as HIV is not adequately managed in PEH resulting in significant downstream healthcare costs in addition to preventable patient suffering. A recent SR identified that housing PEH (in the short term) improves some aspects of health in this population with HIV, anxiety, and depression [229].

However, focusing just on providing housing for this population does not mean that an individual’s health needs are automatically solved. We know from Housing First studies that just providing housing does not result in improvements in mental health or addictions after a year, especially in people who have experienced significant trauma [55]. This umbrella review suggests that the health effects of homelessness are serious, longstanding, and involve all parts of the health system. Finally, while psychological interventions are expected to be more effective than TAU in reducing the burden of the associated health condition among clinical and non-clinical populations [230], the herein reviewed MA focusing on PEH on the matter [220] failed to reach a statistically significant threshold according to our conservative *p* value set at *p*=.005, though it could not be excluded the condition of homelessness itself could attenuate the effects of such psychological interventions.

What is needed now are studies that look at better coordination of care for this population that may involve hospitals and community partners as well as programs to address health issues in people recently housed after experiencing homelessness. In addition, it should be reiterated that there is an urgent need for international standardization of housing status to improve research rigor that could improve the external generalizability of this field and hence direct policy. Furthermore, more studies should be conducted in low- and middle-income countries as the vast majority of studies to date on this topic have been performed in developed nations. Such bias related to the geographical region may depend on a variety of issues, including, but not necessarily limited to, public health policies as well as the limited funding in LMICs. Lastly, more SRs and MAs with enhanced methodological quality are an unmet need in this field.

## Supplementary Information

**Additional file 1: Material 1.** adopted search strings. **Table S1.** criteria for the evaluation of the credibility of the evidence of observational studies. Please note that criterion n.1 (sample size of cases) was purposely waived as outlined in the main-text, methods section. **Table S2.** List of the 179 excluded records, with the reason(s). Note: duplicate records may appear multiple times for consistency issues. **Table S3.** Included SRs or MAs of observational studies; quality rating according to the AMSTAR-2. **Table S4.** Sensitivity analysis of evidence from SRs or MAs of observational studies for the association between homelessness and any health outcome.

## Data Availability

All data generated or analyzed during this study are included in this published article and are available online at https://osf.io/am67d/ and https://osf.io/58mhu/. The additional file includes the following: material 1 – [search strings]; table S1 - [criteria for the evaluation of the credibility of the evidence of observational studies]; table S2 – [excluded studies with reasons]; table S3 – [quality rating of the included SRs or MAs of observational studies, according to the AMSTAR-2]; table S4 – [sensitivity analysis].

## Abbreviations

ADL: Activities of daily living
AMSTAR-2: Assessment of Multiple Systematic Reviews 2
AMSTAR-plus: Assessment of Multiple Systematic Reviews Plus
ART: Antiretroviral therapy
CI: Confidence interval
COVID-19: Coronavirus disease 2019
ES: Effect size
HCV: Hepatitis C virus
HIV: Human immunodeficiency virus
IADL: Instrumental activities of daily living
LMICs: Low- and middle-income countries
MA: Meta-analysis
NOS: Newcastle-Ottawa Scale
PEH: People experiencing homelessness
RCT: Randomized controlled trial
SMR: Standardized mortality ratio
SR: Systematic review
SUD: Substance use disorder
