# Supplementary material for: Homelessness and health-related outcomes: an umbrella review of observational studies and randomized controlled trials
Source: BMC Med. 2022 Jul 12;20:224. doi: 10.1186/s12916-022-02423-z (PMC9273695; doi:10.1186/s12916-022-02423-z)
Supplement: Supplementary file 1 — Additional file 1: Material 1. adopted search strings. Table S1. criteria for the evaluation of the credibility of the evidence of observational studies. Please note that criterion n.1 (sample size of cases) was purposely waived as outlined in the main-text, methods section. Table S2. List of the 179 excluded records, with the reason(s). Note: duplicate records may appear multiple times for consistency issues. Table S3. Included SRs or MAs of observational studies; quality rating according to the AMSTAR-2. Table S4. Sensitivity analysis of evidence from SRs or MAs of observational studies for the association between homelessness and any health outcome. [file 12916_2022_2423_MOESM1_ESM.docx]

**Additional file**

**Material 1:** adopted search strings

PUBMED/MEDLINE Search April 28, 2021 (394 hits)

((((("homeless persons"[MeSH Terms])) OR ("homeless youth"[MeSH Terms])) OR ("vulnerable populations"[MeSH Terms])) OR (homeless*[Title/Abstract])) AND (((("meta analysis as topic"[MeSH Terms]) OR ("systematic reviews as topic"[MeSH Terms])) OR ("meta analysis"[Title/Abstract])) OR ("systematic review"[Title/Abstract]))

EMBASE Search April 28, 2021 (422 hits)

Search #1 homelessness:ti,ab,kw OR 'homeless person':ti,ab,kw OR 'homeless youth':ti,ab,kw OR 'vulnerable population':ti,ab,kw

Search #2 'systematic review topic':ti,ab,kw OR 'meta analysis topic':ti,ab,kw OR 'systematic review':ti,ab,kw OR 'meta analysis':ti,ab,kw

Search #3 #1 AND #2

SCOPUS Search April 28, 2021 (722 hits)

( TITLE-ABS ( "homeles*" ) OR TITLE-ABS ( "vulnerable population" ) ) AND ( TITLE-ABS ( "meta-analysis" ) OR TITLE-ABS ( "systematic review" ) )

**Table S1:** criteria for the evaluation of the credibility of the evidence of observational studies. Please note that criterion n.1 (sample size of cases) was purposely waived as outlined in the main-text, methods section.

| **Classification** | **Criteria** |
| --- | --- |
| Convincing evidence (Class I) | 1. ~~More than 1000 cases~~ 2. Significant summary associations (p<10^-6^) per random-effects calculations 3. No evidence of small-study effects 4. No evidence of an excess of significance bias 5. Prediction intervals not including the null value 6. Largest study nominally significant (p<0.05) 7. Not large heterogeneity (i.e., *I^2^*< 50%) |
| Highly Suggestive evidence (Class II) | 1. ~~More than 1000 cases~~ 2. Significant summary associations (p<10^-6^) per random-effects calculation 3. Largest study nominally significant (p<0.05) |
| Suggestive Evidence (Class III) | 1. ~~More than 1000 cases~~ 2. Significant summary associations (p<10^-3^) per random-effects calculations |
| Weak evidence (Class IV) | 1. All other associations with p≤0.05 |
| Non-significant associations (NS) | 1. All associations with p >0.05 |

**Note**: Strikethrough lines indicate waived criteria.

**Table S2:** List of the 179 excluded records, with the reason(s). Note: duplicate records may appear multiple times for consistency issues.

| (Hino, Yamamoto et al. 2021) | SR without quantitative meta-analytic synthesis of the data |
| --- | --- |
| (Xiang, Kaminga et al. 2021) | Only prevalence provided |
| (Formosa, Kishimoto et al. 2021) | Not enough intervention studies according to our protocol |
| (Ayano, Belete et al. 2021) | Only prevalence provided |
| (Polillo, Gran-Ruaz et al. 2021) | SR without quantitative meta-analytic synthesis of the data |
| (Dalton-Locke, Marston et al. 2020) | Narrative review |
| (Batra, Pharr et al. 2020) | Insufficient number of observational studies according to our protocol |
| (Nevard, Green et al. 2021) | Narrative review; unrelated |
| (Paisi, March-McDonald et al. 2020) | Narrative review; unrelated to our research topic |
| (Sayal, Slomovic et al. 2020) | Only prevalence provided; SR without quantitative meta-analytic synthesis of the data; |
| (Constance and Lusher 2020) | SR without quantitative meta-analytic synthesis of the data |
| (Peng, Hahn et al. 2020) | SR without quantitative meta-analytic synthesis of the data |
| (Chandler, Austin et al. 2020) | SR without quantitative data |
| (Rezaei, Ghiasvand et al. 2020) | Unrelated to our research topic |
| (Ayano, Shumet et al. 2020) | Only prevalence provided |
| (Aubry, Bloch et al. 2020) | Insufficient number of interventional studies according to our protocol |
| (Ayano, Solomon et al. 2020) | Only prevalence provided |
| (Rajendran, Zaki et al. 2020) | Not enough observational studies according to our protocol |
| (Ponka, Agbata et al. 2020) | SR without quantitative meta-analytic synthesis of the data |
| (Carver, Ring et al. 2020) | Unrelated to our research topic |
| (Magwood, Salvalaggio et al. 2020) | A systematic review of systematic reviews |
| (Stubbs, Thornton et al. 2020) | Only prevalence provided |
| (Ayano, Tesfaw et al. 2019) | Only prevalence provided |
| (Wang, Mott et al. 2019) | Insufficient number of interventional studies according to our protocol |
| (Ayano, Tsegay et al. 2019) | Only prevalence provided |
| (Getty, Morande et al. 2019) | Unrelated to our research topic |
| (Riquelme-Miralles, Palazón-Bru et al. 2019) | An insufficient number of interventional studies focused on the same intervention according to our protocol |
| (Gulati, Keating et al. 2019) | Only prevalence provided |
| (Baxter, Tweed et al. 2019) | Insufficient number of interventional studies according to our protocol |
| (Bazzi, Drainoni et al. 2019) | SR without quantitative meta-analytic synthesis of the data |
| (Weber 2019) | An insufficient number of interventional studies focused on the same intervention according to our protocol |
| (Behzadifar, Gorji et al. 2018) | Unrelated to our research topic |
| (Hamilton, Tolfree et al. 2018) | Insufficient number of interventional studies according to our protocol |
| (Heuvelings, Greve et al. 2018) | Insufficient number of interventional studies according to our protocol |
| (Alividza, Mariano et al. 2018) | Insufficient number of observational studies according to our protocol |
| (McPherson, Krotofil et al. 2018) | Narrative review |
| (Klop, de Veer et al. 2018) | SR without quantitative meta-analytic synthesis of the data |
| (Parriott, Malekinejad et al. 2018) | Only prevalence provided |
| (Hanlon, Yeoman et al. 2018) | Insufficient number of interventional studies focused on the same intervention according to our protocol |
| (Gentry, Forouhi et al. 2019) | SR without quantitative meta-analytic synthesis of the data |
| (Silva, Pereira et al. 2018) | Unrelated to our research topic |
| (Ijaz, Thorley et al. 2018) | Insufficient number of observational studies according to our protocol |
| (Luong, Rew et al. 2018) | Only prevalence provided |
| (Degenhardt, Peacock et al. 2017) | Only prevalence provided |
| (Schreiter, Bermpohl et al. 2017) | Only prevalence provided |
| (Hoffberg, Spitzer et al. 2018) | Only prevalence provided |
| (Richter and Hoffmann 2017) | SR without quantitative meta-analytic synthesis of the data |
| (Ijaz, Jackson et al. 2017) | SR without quantitative meta-analytic synthesis of the data |
| (Conn and Ruppar 2017) | No study level data |
| (Heuvelings, de Vries et al. 2017) | SR without quantitative meta-analytic synthesis of the data |
| (To, Brothers et al. 2016) | Only prevalence provided |
| (Fry, Langley et al. 2017) | SR without quantitative meta-analytic synthesis of the data |
| (Moe, Kirkland et al. 2017) | Insufficient number of interventional studies according to our protocol |
| (Sumalinog, Harrington et al. 2017) | Insufficient number of interventional studies according to our protocol |
| (Sandgren, Vonk Noordegraaf-Schouten et al. 2016) | Only prevalence provided |
| (Leibler, Zakhour et al. 2016) | SR without a quantitative meta-analytic synthesis of the data |
| (Health Quality Ontario 2016) | Insufficient number of interventional studies according to our protocol |
| (Ly and Latimer 2015) | Unrelated to our research topic |
| (Thorley, Porter et al. 2015) | Not an original study |
| (Cumber and Tsoka-Gwegweni 2015) | Narrative review |
| (Edlin, Eckhardt et al. 2015) | Only prevalence provided |
| (Benston 2015) | Unrelated to our research topic |
| (Curtis 2016) | Only prevalence provided |
| (Tsai and Rosenheck 2015) | Unrelated to our research topic |
| (Twyman, Bonevski et al. 2014) | Only prevalence provided |
| (Bernstein, Meurer et al. 2015) | Only prevalence provided |
| (Heerde, Scholes-Balog et al. 2015) | Unrelated to our research topic |
| (White and Newman 2015) | No SR or MA |
| (Paquette, Cheng et al. 2014) | Only prevalence provided |
| (Ennis, Roy et al. 2015) | SR without a quantitative meta-analytic synthesis of the data |
| (Roy, Crocker et al. 2014) | Only prevalence provided |
| (McInnes, Li et al. 2013) | Only prevalence provided |
| (Chant, Wang et al. 2014) | No study level data |
| (de Vet, van Luijtelaar et al. 2013) | SR without a quantitative meta-analytic synthesis of the data |
| (Byrne, Montgomery et al. 2013) | SR without a quantitative meta-analytic synthesis of the data |
| (Coren, Hossain et al. 2013) | Unrelated to our research topic |
| (Embleton, Mwangi et al. 2013) | Only prevalence provided |
| (Doran, Ragins et al. 2013) | Narrative review |
| (Woan, Lin et al. 2013) | Duplicate |
| (Tankimovich 2013) | Narrative review |
| (Hodgson, Shelton et al. 2013) | Only prevalence provided |
| (Speirs, Johnson et al. 2013) | Insufficient number of interventional studies according to our protocol |
| (Cassone 2012) | Commentary; not quantitative data |
| (Topolovec-Vranic, Ennis et al. 2012) | SR without quantitative meta-analytic synthesis of the data; only prevalence provided |
| (Beijer, Wolf et al. 2012) | Only prevalence provided |
| (Zlotnick, Tam et al. 2012) | Insufficient number of interventional studies according to our protocol |
| (Fitzpatrick-Lewis, Ganann et al. 2011) | Insufficient number of interventional studies according to our protocol |
| (Bryant, Bonevski et al. 2011) | Insufficient number of interventional studies according to our protocol |
| (Thomas, Gray et al. 2011) | Insufficient number of interventional studies according to our protocol |
| (Borkhoff, Wieland et al. 2011) | No SR or MA |
| (Altena, Brilleslijper-Kater et al. 2010) | SR with no quantitative meta-analytic synthesis of the data |
| (Shortt, Hwang et al. 2008) | SR with no quantitative meta-analytic synthesis of the data |
| (Burra, Stergiopoulos et al. 2009) | SR without a quantitative meta-analytic synthesis of the data |
| (Murray, Bauld et al. 2009) | SR with no quantitative meta-analytic synthesis of the data; not enough intervention studies according to our protocol |
| (Herrman 2008) | No SR or MA |
| (Fazel, Khosla et al. 2008) | Only prevalence provided |
| (Kyle and Dunn 2008) | Narrative review |
| (Schumacher, Milby et al. 2007) | Insufficient number of interventional studies according to our protocol |
| (Leaver, Bargh et al. 2007) | SR without a quantitative meta-analytic synthesis of the data |
| (Coldwell and Bender 2007) | Insufficient number of interventional studies according to our protocol |
| (Parks, Stevens et al. 2007) | SR without a quantitative meta-analytic synthesis of the data |
| (Wright and Walker 2006) | Narrative review |
| (Hwang, Tolomiczenko et al. 2005) | SR without a quantitative meta-analytic synthesis of the data |
| (Spence, Stevens et al. 2004) | SR without a quantitative meta-analytic synthesis of the data |
| (Folsom and Jeste 2002) | Only prevalence provided |
| (Flach and Razza 2021) | SR without a quantitative meta-analytic synthesis of the data |
| (Andrade, Figueiredo et al. 2020) | SR without a quantitative meta-analytic synthesis of the data |
| (Nilsson, Nordentoft et al. 2020) | Unrelated to our research topic |
| (Muzzey, Fortenberry et al. 2020) | Unrelated to our research topic |
| (Omerov, Craftman et al. 2020) | SR without a quantitative meta-analytic synthesis of the data |
| (Hamilton, Tolfree et al. 2019) | Unrelated to our research topic |
| (Formosa, Kishimoto et al. 2019) | Full-text not available |
| (Heerde and Hemphill 2019) | Unrelated to our research topic |
| (Blanquet, Legrand et al. 2019) | Unrelated to our research topic |
| (Charron, Hayes et al. 2018) | Full-text not available |
| (Kenworthy, Ayyub et al. 2017) | Unrelated to our research topic; full-text not available |
| (Whitney, Kruszka et al. 2017) | No MA or SR |
| (Simmonds, Simmonds et al. 2016) | Only prevalence provided; full-text not available |
| (Schneider, Skaathun et al. 2015) | No MA or SR |
| (Abad, Baack et al. 2014) | SR without a quantitative meta-analytic synthesis of the data |
| (Auerswald, Woan et al. 2013) | SR without a quantitative meta-analytic synthesis of the data |
| (Chant, Smith et al. 2013) | No SR or MA |
| (Chant, Smith et al. 2013) | Full-text not available |
| (da Silva, Lovisi et al. 2012) | SR without a quantitative meta-analytic synthesis of the data; unrelated to our research topic |
| (Coufopoulos, McDowell et al. 2012) | Not available full text |
| (Wilson, Aidala et al. 2012) | Narrative review |
| (Sundin 2011) | No SR or MA |
| (Altena, Brilleslijper-Kater et al. 2010) | SR without quantitative meta-analytic synthesis of the data |
| (Moniruzzaman, Kazanjian et al. 2010) | Only prevalence provided |
| (Wright and Walker 2006) | Narrative review (a duplicate record) |
| (Lowrie, Stock et al. 2021) | No SR or MA |
| (Ly, Castaneda et al. 2021) | Only prevalence provided |
| (Hino, Yamamoto et al. 2021) | Only prevalence provided |
| (Vijayaraghavan, Elser et al. 2020) | Insufficient number of interventional studies according to our protocol |
| (Morton, Kugley et al. 2020) | Unrelated to our research topic |
| (Ayano, Ayano et al. 2020) | Only prevalence provided |
| (Hossain, Purohit et al. 2020) | Umbrella Review |
| (Magwood, Salvalaggio et al. 2020) | Umbrella Review |
| (Brownfield, Thielking et al. 2020) | Unrelated to our research topic |
| (Paisi, March-Mcdonald et al. 2020) | Narrative review |
| (Soar, Dawkins et al. 2020) | Only prevalence provided |
| (Yadee, Bangpan et al. 2019) | Unrelated to our research topic |
| (Dorney-Smith, Thomson et al. 2019) | Non systematic review |
| (Bazzi, Drainoni et al. 2019) | Unrelated to our research topic |
| (Mendes, Ronzani et al. 2019) | SR without quantitative meta-analytic synthesis of the data |
| (Noh 2018) | No study level data |
| (El Baba and Colucci 2018) | Unrelated to our research topic |
| (Krahn, Caine et al. 2018) | Insufficient number of interventional studies according to our protocol |
| (Silva, da Silva Pereira et al. 2018) | Unrelated to our research topic |
| (Schreiter, Bermpohl et al. 2017) | Only prevalence provided |
| (Heerde and Hemphill 2017) | Unrelated to our research topic |
| (Hoell, Franz et al. 2017) | SR without quantitative meta-analytic synthesis of the data |
| (Campos-Matos, Russo et al. 2016) | Unrelated to our research topic |
| (Adams-Guppy and Guppy 2016) | SR without quantitative meta-analytic synthesis of the data |
| (Polcin 2016) | Narrative review |
| (Costa, Hagan et al. 2015) | Unrelated to our research topic |
| (Martinez, Wu et al. 2015) | Unrelated to our research topic |
| (Heerde and Hemphill 2015) | Unrelated to our research topic |
| (Roy 2014) | Unrelated to our research topic |
| (Chant, Wang et al. 2014) | SR without quantitative meta-analytic synthesis of the data |
| (Heerde and Hemphill 2014) | Unrelated to our research topic |
| (Sudarsanam and Tharyan 2014) | Unrelated to our research topic |
| (De Vet, Van Luijtelaar et al. 2013) | Insufficient number of interventional studies according to our protocol |
| (Hodgson, Shelton et al. 2013) | Only prevalence provided |
| (Momsen, Rasmussen et al. 2012) | Narrative review |
| (Scappaticci and Blay 2010) | Narrative review |
| (Bonner and Luscombe 2009) | Duplicate |
| (Banks, McHugo et al. 2002) | Unrelated to our research topic |
| (Bahji and Bajaj 2018) | Unrelated to our research topic |
| (Clark, Lintzeris et al. 2002) | Unrelated to our research topic |
| (Barajas-Nava 2017) | Unrelated to our research topic |
| (Karki, Shrestha et al. 2016) | Unrelated to our research topic |
| (Mattick, Breen et al. 2014) | Unrelated to our research topic |
| (Mattick, Breen et al. 2009) | Unrelated to our research topic |
| (Saulle, Vecchi et al. 2017) | Unrelated to our research topic |
| (Simoens, Matheson et al. 2005) | Unrelated to our research topic |
| (Strang, Groshkova et al. 2015) | Unrelated to our research topic |
| (Thomas, Fullerton et al. 2014) | Unrelated to our research topic |
| (Maglione, Raaen et al. 2018) | Unrelated to our research topic |

**Table S3:** Included SRs or MAs of observational studies; quality rating according to the AMSTAR-2.

| **Author, year** | **AMSTAR-2 1** | **AMSTAR-2 2** | **AMSTAR-2 3** | **AMSTAR-2 4** | **AMSTAR-2 5** | **AMSTAR-2 6** | **AMSTAR-2 7** | **AMSTAR-2 8** | **AMSTAR-2 9** | **AMSTAR-2 10** | **AMSTAR-2 11** | **AMSTAR-2 12** | **AMSTAR-2 13** | **AMSTAR-2 14** | **AMSTAR-2 15** | **AMSTAR-2 16** |
| --- | --- | --- | --- | --- | --- | --- | --- | --- | --- | --- | --- | --- | --- | --- | --- | --- |
| Aidala et al., 2016 | NO | PARTIAL YES | NO | NO | YES | NO | NO | PARTIAL YES | PARTIAL YES | NO | NA | NA | YES | NO | NA | NO |
| Aldridge et al., 2017 | YES | NO | NO | NO | NO | YES | NO | NO | NO | NO | NO | NO | NO | NO | NO | YES |
| Al-Shakarchi et al., 2020 | YES | NO | NO | NO | YES | NO | NO | PARTIAL YES | PARTIAL YES | NO | NO | NO | YES | NO | NO | NO |
| Arum et al., 2021 | YES | PARTIAL YES | NO | PARTIAL YES | YES | YES | NO | PARTIAL YES | YES | NO | YES | YES | NO | YES | YES | YES |
| Bassuk et al., 2015 | NO | YES | NO | NO | YES | YES | NO | PARTIAL YES | NO | NO | NO | NO | NO | YES | NO | YES |
| Lin et al., 2019 | NO | NO | NO | NO | NO | YES | NO | PARTIAL YES | PARTIAL YES | NO | YES | NO | YES | YES | YES | YES |
| Suh et al., 2020 | YES | NO | NO | NO | YES | YES | NO | NO | NO | NO | NO | NO | NO | NO | NO | YES |
| Tweed et al., 2021 | YES | YES | NO | PARTIAL YES | YES | YES | NO | NO | YES | NO | NO | NO | NO | NO | NO | NO |
| van Draanen et al., 2020 | YES | YES | NO | NO | NO | NO | NO | PARTIAL YES | YES | NO | NA | NA | YES | NO | NA | YES |

Legend for Table S3: MA=Meta-analysis; NA=Not applicable; SR=Systematic Review; AMSTAR-2=A Measurement Tool to Assess Systematic Reviews, 2^nd^ revision.

Please note: The underlined items of the AMSTAR-2 represent critical assessment. Please refer to: <https://amstar.ca/docs/AMSTAR-2.pdf> for additional guidance for each item.

**Table S4:** Sensitivity analysis of evidence from SRs or MAs of observational studies for the association between homelessness and any health outcome.

|  | | | | | | | **Criteria for Level-of-Evidence Classification** | | | | |  | |
| --- | --- | --- | --- | --- | --- | --- | --- | --- | --- | --- | --- | --- | --- |
| **Author, year** | **Adverse health outcomes** | **Exposed/**  **Unexposed** | **Prevalence (%) based on high-quality cohort studies** | **No. of included studies per association** | **Random effects**  **measure, ES (95% CIs)** | **Results** | **p-value**  **random**  **effects** | **I^2^ (p-value)** | **PIs (95 % CIs)** | **SSE/ESB** | **LS** | **Change of CE after sensitivity** | **AMSTAR-2 quality** |
| Good Quality Prospective-Cohort studies according to NOS | | | | | | | | | | | | | |
| Aidala et al., 2016 | Hospitalization due to any cause | Homeless with HIV/Non-homeless with HIV | 21.8% | 3 | OR: 1.99 (1.58-2.49) | Increased chances for Homeless with HIV | 2.5x10^-9^ | 4.88% (0.749) | 0.41-9.64 | NO/NO | YES | I to II | CRITICALLY LOW |
| Aldridge et al., 2017 | Mortality due to any cause | Homeless/General population | NA | 2 | SMR: 6.11 (5.14-10.58) | Increased chances for Homeless | 2.7x10^-94^ | 95.06% (0.0001) | NA | NA/NA | YES | No Change (II) | CRITICALLY LOW |
| Aldridge et al., 2017 | Mortality due to external causes, as defined by ICD-10 | Homeless/General population | NA | 6 | SMR: 15.75 (10.58-23.44) | Increased chances for Homeless | 5.2x10^-42^ | 97.43% (0.0001) | 3.63–68.17 | NO/NA | YES | No Change (II) | CRITICALLY LOW |
| Tweed et al., 2021 | Mortality due to any cause | Homeless with SUD/Homeless without SUD | 48.5% | 3 | HR: 2.1 (1.57-2.82) | Increased chances for Homeless with SUD | 6.3x10^-7^ | 90.3% (0.0001) | 0.06-79.05 | YES/NO | YES | NS to II | CRITICALLY LOW |
| Aidala et al., 2016 | Emergency department use due to any cause | Homeless with HIV/Non-homeless with HIV | 18.39% | 2 | OR: 1.8 (1.4-2.32) | Increased chances for Homeless with HIV | 3.8x10^-6^ | 27.59% (0.297) | NA | NA/NO | YES | No Change (III) | CRITICALLY LOW |
| Al-Shakarchi et al., 2020 | Diagnosis of cardiovascular disease, as defined by ICD-10 | Homeless/Non-homeless | NA | 2 | OR: 4.25 (2.03-8.9) | Increased chances for Homeless | 1.2x10^-4^ | 84.3% (0.008) | NA | NA/NA | YES | No Change (III) | CRITICALLY LOW |
| Aidala et al., 2016 | Nonadherence to antiretroviral therapy | Homeless/Non-homeless | 8.5% | 2 | OR: 1.65 (1.16-2.36) | Increased chances for Homeless | 0.005 | 4.9% (0.555) | NA | NA/YES | YES | III to IV | CRITICALLY LOW |
| Tweed et al., 2021 | Mortality due to external causes, as defined in ICD-10 | Homeless with SUD/Homeless without SUD | NA | 4 | HR: 2.3 (1.26-4.2) | Increased chances for Homeless with SUD | 0.0069 | 78.5% (0.093) | 0.17-31.02 | NO/NA | YES | No Change (IV) | CRITICALLY LOW |
| Aidala et al., 2016 | Mortality due to any cause | Homeless with HIV/Non-homeless with HIV | 26.4% | 2 | HR: 1.65 (0.63-4.33) | Increased chances for Homeless with HIV | 0.305 | 76.5% (0.031) | NA | NA/NO | NO | No Change (NS) | CRITICALLY LOW |
| Tweed et al., 2021 | Mortality due to any cause | Homeless with SMI/Homeless without SMI | 8.3% | 2 | HR: 0.86 (0.67-1.1) | Increased chances for Homeless without SMI | 0.236 | 18.9% (0.37) | NA | NA/NP | NO | No Change (NS) | CRITICALLY LOW |
| Tweed et al., 2021 | Mortality due to external causes, as defined by ICD-10 | Homeless with SMI/Homeless without SMI | NA | 3 | HR: 3.13 (0.78-12.51) | Increased chances for Homeless with SMI | 0.106 | 93.4% (0.003) | 0.001-9.6x10^7^ | NO/NA | YES | No Change (NS) | CRITICALLY LOW |

Legend for Table S4: CE=Credibility evidence; CI=Confidence Interval; ES=Effect size; ESB=Excess significance bias; HR=Hazard Ratio; ICD-10=International Classification of Diseases, 10^th^ revision; LS=Largest study with significant effect; MA=Meta-analysis; NA=Not applicable; NOS=Newcastle-Ottawa Scale; NP=Not pertinent because of fewer than expected number.
